# The First Step of Health Policy-Making for Lifestyle Modifications at Middle Age: Problem Identification in 40- to 60-Year-Old Population, Northern Iran

**DOI:** 10.1155/2018/9895346

**Published:** 2018-07-16

**Authors:** Simin Mouodi, Seyed Reza Hosseini, Reza Ghadimi, Ali Bijani, Robert Graham Cumming, Hassan Ashrafian Amiri, Fatemeh Bayani, Shima Sum

**Affiliations:** ^1^Social Determinants of Health Research Center, Health Research Institute, Babol University of Medical Sciences, Babol, Iran; ^2^Health Research Institute, Babol University of Medical Sciences, Babol, Iran; ^3^School of Public Health, University of Sydney, Sydney, NSW, Australia

## Abstract

**Background:**

The purpose of this study was to determine the current situation of lifestyle behaviors and related outcomes, as the first step to make proper local health policies for improvement of health lifestyle behaviors.

**Materials and Methods:**

This analytic research has been conducted as a cross-sectional study on the middle-aged (40–60 years old) population of Amirkola, Northern Iran. The Persian translation of the Health-Promoting Lifestyle Profile II questionnaire and the International Physical Activity Questionnaire were used for data collection. Also, anthropometric variables, blood pressure, fasting blood sugar, and serum lipids profile were examined.

**Results:**

Three hundred one individuals have been enrolled in the study. Results showed that 10.6% of the participants had unhealthy lifestyle behaviors in the subdomain of spiritual growth; 46.8% in the subdomain of health responsibility; and 48.2% in the subdomains of stress management, physical activity, and nutrition; men had more physical activity than women (*p* < 0.001). Totally, 189 persons (63.9%) had serum cholesterol greater than or equal to 200 mg/dL; seventy-five individuals (25%) had high blood pressure. One hundred twenty-six persons (81.8%) of women and 103 (70.1%) of men were overweight or obese.

**Conclusions:**

Health lifestyle behaviors in 40- to 60-year-old population need a proper intervention to improve the current situation.

## 1. Introduction

The increasing prevalence of noncommunicable diseases (NCDs), especially cardiovascular diseases, diabetes, cancer, and chronic respiratory disorders, has driven health policy-makers to implement effective programs for prevention and control of these diseases, worldwide [1–3]. These major NCDs share four behavioral risk factors which represent one's lifestyle, namely, tobacco use, unhealthy diet, physical inactivity, and harmful use of alcohol [4]. As lifestyles become more sedentary and diets change more rapidly than ever, these diseases are becoming more common and are striking at a younger age [5]. Recently, greater efforts have been made to best encourage people to have an active living and healthier lifestyle, including making better nutritional patterns and physical activity (PA) choices [6–13]. The World Health Organization (WHO) advocates to all countries, either developed or developing, to make sustainable policies for fighting NCDs, as the most important health threats [1]. The WHO reported that NCDs kill 16 million people each year before their 70th birthdays [1]. Unhealthy diet and lack of physical activity are leading global risks to health [14]. Globally, 1 in 4 adults is not active enough [15]. Worldwide obesity has more than doubled since 1980; in 2014, more than 1.9 billion adults, 18 years and older, were overweight; of these, over 600 million were obese. Thirty-nine percent of adults aged 18 years and above were overweight in 2014, and 13% were obese [16].

Recent studies predicted that disability-adjusted life years related to cardiovascular diseases would be increased more than twofold in 2025 compared with 2005 in Iranian adults aged ≥30 years [17]. The Isfahan Cohort Study conducted in the central part of Iran showed that the incidence rates for almost all cardiovascular diseases were high in adults aged 35 years and above [18]. According to the third national surveillance of risk factors of noncommunicable diseases in Iranian adults (aged 15–64 years), the prevalence of diabetes, hypertension, obesity, central obesity, hypertriglyceridemia, and hypercholesterolemia was reported to be 8.7%, 26.6%, 22.3%, 53.6%, 36.4%, and 42.9%, respectively [19]; furthermore, the prevalence of low physical activity was estimated to be 44.8%, and the odds of lower physical activity in the women were three times greater than those in men [20]. The survey of Tehran Lipid and Glucose Study in adult population (aged 30–74 years) over 6 years of follow-up suggested that current situation of noncommunicable disease risk factors needs a proper lifestyle intervention regarding changes in nutritional habits and increased physical activity for primary prevention of cardiovascular diseases in Iranian adults [21, 22].

Considering the importance of novel, local, cost-effective, and culturally acceptable interventions to change sedentary lifestyle [23–26] and due to limited published articles related to the adults' lifestyle behaviors in the northern region of Iran, this study aimed to evaluate the current situation of health-promoting lifestyle profile, physical activity, anthropometric variables, and the profile of plasma lipids and glucose in the middle-aged population of Northern Iran, near Caspian Sea. The results can be used as the first step of policy-making to implement proper interventions [12, 27] for lifestyle modifications in this region.

## 2. Materials and Methods

This analytic research has been conducted as a cross-sectional study on the middle-aged (40–60 years old) population of Amirkola, Northern Iran. Sample size was determined based on data from the previous studies by considering *p*=0.5 and *d*=0.06 for a sample comprising 267 individuals [28]; in addition, taking a possible loss to follow-up into account, a safety margin of 10–15% was used [29]. Considering that the entire population of this area is covered by the family physician program, we used various methods to invite and recruit middle-aged population, including (1) informing local health care workers and family physicians; (2) public announcements by local health volunteers (nonemployed women who have voluntary cooperation with health centers); (3) distribution of public notices in households, public gathering places, mosques, banks, post offices, local markets, and stores; and (4) selecting the research center in the central part of the city, close to the local health centers, in the access of people of the region and keeping this center active at various times of the morning and evening on Saturdays to Thursdays each week in order to be responsive to the clients. At first, the volunteers who referred to the research center have been checked for exclusion criteria; then, they were divided into 4 groups according to their sex and age (male and female; in the age groups of 40–49 and 50–60 years); at the same time, using the information available about households which were associated with the health centers of the region, the researchers called the households which had at least one person in the age group of 40–60 years and invited them to participate in the study. Simple random sampling was used to select these households. Sampling continued until each of the four groups had nearly the same number of participants. Women in the pregnancy or breast-feeding period, the persons who could not read and write (and could not read the written informed consent form of the study), and those who were undergoing specific nutrition regimens, prescribed by a physician, have been excluded.

The Persian translation of the Health-Promoting Lifestyle Profile II (HPLP-II) questionnaire was used to investigate participants' lifestyles. This questionnaire includes 52 questions in six lifestyle subscales (spiritual growth with “11 questions,” health responsibility “13 questions,” interpersonal relations “8 questions,” stress management “6 items,” physical activity “7 questions,” and nutritional habits “7 questions”). Each question has a four-point response scale to determine the frequency of that behavior, ranging from 1–4: “1” representing “never” and “4” representing “routinely.” An average of ≥2.50 was considered to be a positive response in each question [30]; therefore, considering the number of questions in each subdomain, the cutoff point for health behaviors related to spiritual growth was considered as 27.5, health responsibility as 32.5, interpersonal relations as 20, stress management as 15, physical activity, and nutritional habits as 17.5. The HPLP-II instrument has been translated in different languages [31, 32]; validity and reliability of its Iranian version have been confirmed in previous studies [33, 34].

We used the International Physical Activity Questionnaire (IPAQ) to assess physical activity (PA) of the participants. Validity and reliability of its Iranian version have been confirmed in the year 2012 [35]. This questionnaire includes four parts ((1) activities at work; (2) housework, gardening, and caring for family; (3) PA in transportation; and (4) recreation, sport, and leisure time) and asks about the intensity and time a person spent being physically active in the last 7 days. For each domain, participants recorded the number of days and time spent each day undertaking vigorous- and moderate-intensity activities separately along with the time spent walking. Vigorous physical activities are defined as activities that take hard physical effort and make a person breathe much harder than normal such as lifting heavy things, digging, heavy construction work, or climbing stairs, and moderate activities are defined as activities that take moderate physical effort and make them breathe somewhat harder than normal such as carrying light loads. These values (vigorous activity, moderate activity, and walking) were used to calculate the PA levels, as specified in the official IPAQ instruction manual. Each type of activity was weighted by its energy requirements as MET (metabolic equivalent of task). By multiplying MET value of particular activity (3.3 METs for walking, 4.0 METs for moderate PA, and 8.0 METs for vigorous PA), total MET minutes per week was calculated. Vigorous and moderate activities were defined as those lasting for at least 10 minutes continuously [35]. Questions related to time spent sitting on a weekday or weekend were excluded from analysis. Study population was classified into the levels of PA according to MET minutes/week: “minimally active” for individuals who achieved a minimum of at least 600 MET minutes/week, “highly active” for those who had a minimum of at least 1500 MET minutes per week, and “inactive” persons for those who had not met the above two categories [36].

Other data which were collected included age, gender, education level, occupation, marital status, living region (urban or rural), and history of comorbid physical or mental disorders. Also, weight and height, blood pressure, fasting blood sugar, and serum lipids profile were examined. Body mass index (BMI) was calculated as weight (in kilograms)/height^2^ (in meters).

Other anthropometric measures which have been assessed included body fat percentage and waist and buttock circumferences. These variables have been measured with a tape. Also, waist-to-hip ratio (WHR) and waist-to-height ratio (WHtR) [37, 38] have been calculated.

Waist circumference was measured around the midpoint between the lower margin of the last palpable rib and the top of the iliac crest, and buttock measurement was taken at the maximum circumference over the buttocks. All measurements were taken once over light clothing, and values were recorded in centimeters.

Body fat percentage was measured by using the hand-to-foot bioelectrical impedance analysis technique with a digital body fat calculator (BF511 model; Omron company). This technique has been reported as a simple, quick, and noninvasive method which can give reliable measurements of body composition with minimal intra- and interobserver variability; the results are available immediately and reproducible with <1% error on repeated measurements [39].

Blood pressure was measured with the participant in the sitting position, using a digital sphygmomanometer (Omron M6 brand), and the average of two times blood pressure measurement ≥140/90 mmHg was defined as hypertension. Early morning venous blood samples (5 mL) were collected after fasting for at least 12 hours to assess fasting blood sugar (FBS), total cholesterol, high-density lipoprotein (HDL) cholesterol, low-density lipoprotein (LDL) cholesterol, and triglyceride (TG) levels; these values were measured using Pars Azmoon kits via the autoanalyzer respons®910 DiaSys system. All of the laboratory tests have been conducted in a particular laboratory which had undertaken external quality control.

According to the National Cholesterol Education Program Adult Treatment Panel III Report [37], cutoff points for fasting blood glucose, total serum cholesterol, triglyceride, and LDL levels have been considered less than 126, 200, 150, and 100 mg/dL, respectively. The HDL cholesterol cutoff point was considered less than 40 mg/dL in men and less than 50 mg/dL in women. Also, serum TG levels in the range of 150–199 were classified as borderline-high TG, 200–499 as high TG, and >500 mg/dL as very high TG categories.

Data analysis was performed by SPSS 17 package; the chi-square test, Fisher's exact test, and *t*-test were used for data analysis with a significance level of *p* < 0.05. The Kolmogorov–Smirnov test was used to evaluate the normal distribution of quantitative data.

All of the participants signed a written informed consent form. They have been assured that their information would be kept confidential. This research has been approved by the Ethics Committee of Babol University of Medical Sciences as registration code Mubabol.Rec.1394.45.

## 3. Results

Three hundred one individuals have been enrolled in this study. The mean age of the participants was 49.67 ± 5.42 (range of 40–60 years); 154 persons (51.2%) were female with the mean age of 49.0 ± 5.13 years, and 147 (48.8%) were male with the mean age of 50.4 ± 5.64. Basic characteristics of the participants divided into the two age groups are presented in Table 1. This table shows that these two age groups had no significant difference in their baseline characteristics (*p* > 0.05), except in their occupation (*p*=0.047).

Lifestyle behaviors according to the HPLP-II questionnaire are presented in Table 2. Thirty-two participants (10.6%) in the subdomain of spiritual growth, 141 participants (46.8%) in the subdomain of health responsibility, 145 participants (48.2%) in the subdomain of interpersonal relations, and 145 participants (48.2%) in the subdomains of stress management, physical activity, and nutrition had scores less than average. There was no significant difference between the two age groups about distribution of the average score of lifestyle behaviors except in the nutrition subscale (*p*=0.033); the age group of 50–60 years had higher scores in this subscale. Men and women had no significant difference in the total score and subdomains of lifestyle behavior (*p* > 0.05) except in the subdomain of health responsibility (*p* < 0.001); in this subscale, women had higher scores.

Distribution of PA in two sexes divided into the two age groups is presented in Table 3. Totally, 24 women (15.6%) and 1 man (0.7%) were inactive, 36 women (23.4%) and 13 men (8.8%) were minimally active, and 94 women (61.0%) and 133 men (90.5%) were highly active according to MET min/week. A significant statistical difference was observed between two sexes about their physical activity (*p* < 0.001); men had more physical activity than women.

Anthropometric measures, blood pressure, fasting blood sugar, and serum lipids profile of the participants divided into two age groups are presented in Table 4. This table shows that these two age groups have significant differences in height, waist circumference, WHR, WHtR, and systolic blood pressure (*p* < 0.05). We found significant differences in mean systolic blood pressure, BMI, body fat percentage, waist and buttock circumferences, WHR, WHtR, fasting blood glucose, and TG and HDL serum levels between male and female persons (*p* < 0.05). Mean systolic blood pressure and waist circumference, WHR, WTR, fasting blood glucose, and TG serum level were higher in men; BMI, body fat percentage, buttock circumference, WHtR, and HDL were higher in women.

In this research, we found 19 individuals (6.4%) with FBS greater than or equal to 126 mg/dL: 5 (3.3%) of women and 14 (9.7%) of men; 189 persons (63.9%) with serum cholesterol greater than or equal to 200 mg/dL: 93 (61.6%) of women and 96 (66.2%) of men; 58 persons (19.6%) with borderline-high TG, 79 persons (26.7%) with high TG; and 6 persons (2.0%) with very high TG levels. One hundred thirteen (74.3%) of women and 126 (86.9%) of men had normal HDL levels (*p*=0.006), and 85 persons (28.6%) had LDL cholesterol less than or equal to 100 mg/dL. Fifty-one subjects (17.0%) had systolic blood pressure greater than or equal to 140 mmHg, 55 persons (18.3%) had diastolic blood pressure greater than or equal to 90 mmHg, and totally 75 individuals (25%) had high blood pressure.

**Figure 1:**
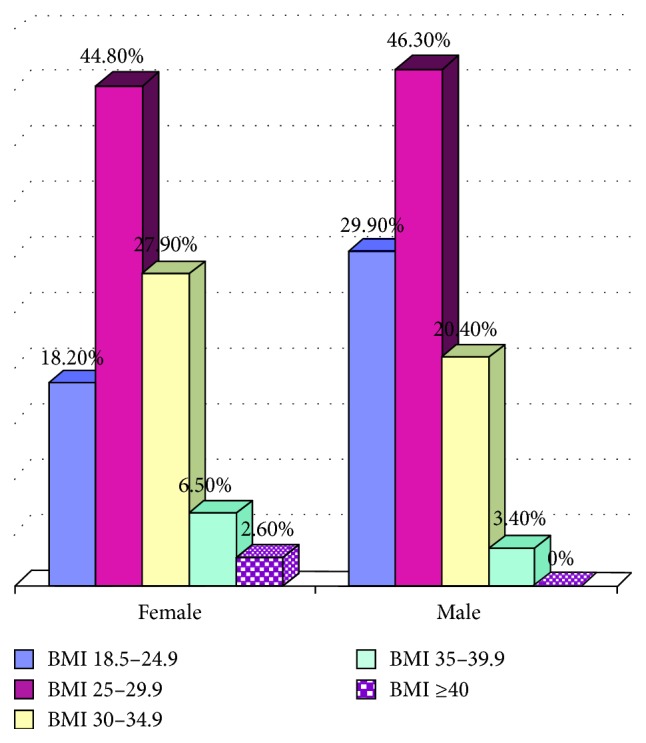
Body mass index distribution in two sexes.

Relative distribution of body mass index categories in two sexes is presented in Figure 1. Nobody has BMI less than 18.5 kg/m^2^ in this study. One hundred twenty-six persons (81.8%) of women and 103 (70.1%) of men were overweight or obese.

## 4. Discussion

We found that 10.6% of 40- to 60-year-old adults had unhealthy lifestyle behaviors in the subdomain of spiritual growth; 46.8% in the subdomain of health responsibility; and 48.2% in the subdomains of stress management, physical activity, and nutrition. This finding shows that interventional approaches for improvement of health-promoting behaviors in this region should primarily focus on stress management skills, physical activity, and healthy nutrition program. In the research performed by Enjezab et al. in Yazd, the central part of Iran, in which 483 middle-aged women were assessed, spiritual growth and physical activity subdomains had the highest and the lowest scores, respectively [40]; the research done by Mirghafourvand et al. in Tabriz, the northwest part of Iran, in which 322 infertile couples were examined, showed that the highest mean score was for the nutrition subscale and the lowest was for physical activity and health responsibility subscales in both men and women [41]; in another research conducted by Mirghafourvand et al., women in reproductive age scored highest in interpersonal relations and lowest in physical activity [42]. In the research carried out by Kim et al. in the United Arab Emirates which evaluated health-promoting lifestyle behaviors among Arabs and Koreans, spiritual growth and interpersonal relations had the highest scores; Arabs scored low on physical activity, and Koreans scored low on health responsibility [43]. These differences in health lifestyle behaviors can be related to ethnic characteristics, cultural variations, and the properties of the population under study which can have impact on the results.

In our study, the 50- to 60-year-old participants had higher scores in the nutrition subscale of the HPLP-II questionnaire; also, women had higher scores in the subdomain of health responsibility. These results show that we should pay more attention to dietary patterns of young adults and should implement interventional plans to encourage men to improve their health care. Okubo et al. reported in their study that older people tended to pay more attention to their diets than younger adults [44]. Vlassoff reported that gender is an important factor which can influence the determinants and consequences of health and illness. He suggested the involvement of both men and women in health education and interventions for promotion of health lifestyles [45], and Bertakis et al. reported that women had a significantly higher number of visits to their primary care clinic and diagnostic services than men. In their study, mean charges for primary care, specialty care, emergency treatment, and diagnostic services and annual total charges were higher for women than men [46].

In this study, nearly 16% of women were inactive and 23.4% of them were minimally active, and men had more physical activity than women. The World Health Organization recommends that adults aged 18–64 should do at least 150 minutes of moderate-intensity aerobic PA or do at least 75 minutes of vigorous-intensity PA throughout the week or an equivalent combination of moderate- and vigorous-intensity activity. This organization advices that, for additional health benefits, adults should increase their moderate-intensity PA to 300 minutes per week or engage in 150 minutes of vigorous-intensity aerobic PA per week [47]; therefore, considering the results, we should implement interventional programs to improve PA in 40- to 60-year-old population, especially for women. In the research done by Asgari et al. in Iran, 32.5% of study population aged 15–64 years did exercise at least 10 minutes in their free time [48], and in the research performed by Jurakić et al., conducted on 1032 Croatians aged 15 years and above, the lowest physical activity was found in the 15–24 age group (42.7 MET hours/week) and the highest in the 55–64 age group (72.0 MET hours/week); they reported that the majority of participants (74%) reached the level of at least 30 minutes of moderate physical activity 5 days a week, which is considered as the lowest level of physical activity for achieving health benefits [49]. In a systematic review conducted to explore the PA patterns among South Asian adults, the overall prevalence of inactivity was reported as follows: India 18.5%–88.4%, Pakistan 60.1%, and Sri Lanka 11.0%–31.8% [50]. In the research of Anjana et al. which was conducted among individuals aged  ≥20 years in four regions of India, similar to the results of our study, men were significantly more active than women [51]. Yates et al. evaluated differences in levels of PA between white and South Asian populations in Leicestershire, UK. They showed that South Asian participants self-reported less moderate-intensity to vigorous-intensity PA (30 versus 51 min/day) and walking (11 versus 17 min/day) [52]. Considering a wide variation in the prevalence of physical inactivity within and between countries [50], these differences in the results can be justifiable.

Mean systolic blood pressure, waist circumference, WHR, WTR, fasting blood glucose, and TG serum level were higher in men; BMI, body fat percentage, buttock circumference, WHtR, and HDL were higher in women. In this research, 6.4% of the participants had FBS ≥126 mg/dL; 63.9% had serum cholesterol ≥200 mg/dL; and 19.6% had borderline-high TG, 26.7% high TG, and 2.0% had very high TG levels. Nine percent of study population had HDL <40 mg/dL, and nearly 29% had LDL ≤100 mg/dL. Almost twenty-five percent of the persons had blood pressure ≥140/90 mmHg. Seventy-six percent of 40- to 60-year-old adults were overweight or obese (BMI ≥ 25).

In the research of Asgari et al. in Iran, 7.7% were diabetics and 25.2% were hypertensive at the age of 25–64 years. Obesity, overweight, and hypercholesterolemia were 14.8, 28.6, and 15.1, respectively, at the age of 15–64 years [48]. In the research of Bakhshi et al. in Iran, 22.3% of the adults aged ≥15 years in all provinces of this country were reported obese. Among all racial/ethnic groups, the highest prevalence value was observed in Turk/Gilak/Lor/Turkmen Iranian ethnicities group (25.5%) [53]. In the research of Krishna Kumar Aryal in Nepal among 15- to 69-year-old population, 21% were overweight or obese. The prevalence of raised blood pressure and raised blood glucose including those on medication was 26% and 4%, respectively; also, almost 23% of study population had raised total cholesterol (total cholesterol ≥190 mg/dL or under current medication for raised cholesterol). The World Health Organization advices to all countries to implement proper policies to reduce modifiable risk factors for NCDs and underlying social determinants through creation of health-promoting environments [4]. We should have policy options in our region to improve health lifestyle behaviors and control the current situation of NCD-related risk factors.

The most important strength of our research was evaluation of NCD-related risk factors in a population-based sample.

We did not take into account some of the behaviors such as alcohol, tobacco, and substance abuse which we did not intend to enter in this study, and this can be considered as a limitation of this research.

## 5. Conclusion

Health lifestyle behaviors in 40- to 60-year-old population need a proper intervention to improve the current situation.

## Figures and Tables

**Table 1:** Basic descriptive characteristics of the participants.

Variables	Age groups		Total (*N*=301), *n* (%)	*p* value
	40–49 years (*N*=145), *n* (%)	50–60 years (*N*=156), *n* (%)		
*Education level*				
Less than diploma	88 (60.7)	89 (57.1)	177 (58.8)	0.408
Diploma	37 (25.5)	50 (32.1)	87 (28.9)	
Higher than diploma	20 (13.8)	17 (10.9)	37 (12.3)	

*Occupation*				
Shopkeepers	7 (4.8)	5 (3.2)	12 (4.0)	0.047
Housewives	72 (49.7)	63 (40.4)	135 (44.9)	
Employees	18 (12.4)	31 (19.9)	49 (16.3)	
Others	48 (33.1)	57 (36.5)	105 (34.9)	

*Marital status*				
Married	140 (96.6)	146 (93.6)	286 (95.0)	0.066
Single, divorced, or widowed	5 (3.4)	10 (6.4)	15 (5.0)	

*Living region*				
Urban	142 (97.9)	150 (96.2)	292 (97.0)	0.288
Rural	3 (2.1)	6 (3.8)	9 (3.0)	

*Past medical history of physical disorders*				
No	118 (81.4)	117 (75.0)	235 (78.1)	0.181
Yes	27 (18.6)	39 (25.0)	66 (21.9)	

*Past medical history of mental disorders*				
No	142 (97.9)	146 (93.6)	288 (95.7)	0.064
Yes	3 (2.1)	10 (6.4)	13 (4.3)	

**Table 2:** Distribution of the average score of lifestyle behaviors according to the HPLP-II questionnaire.

Lifestyle subdomains	Mean score of lifestyle subdomains in two age groups		Total mean ± SD (min–max)	*p* value
	40–49 years (*N*=145)	50–60 years (*N*=156)		
Spiritual growth	33.81 ± 5.31	33.81 ± 4.98	33.81 ± 5.13 (16–44)	0.999
Health responsibility	34.11 ± 7.38	32.70 ± 7.34	33.38 ± 7.38 (16–51)	0.097
Interpersonal relations	23.87 ± 4.57	23.76 ± 4.00	23.81 ± 4.27 (10–32)	0.820
Stress management	13.76 ± 3.07	13.59 ± 2.88	13.67 ± 2.97 (7–22)	0.622
Physical activity	12.99 ± 4.67	13.03 ± 4.84	13.01 ± 4.75 (7–28)	0.934
Nutrition	20.12 ± 3.32	20.63 ± 3.04	20.38 ± 3.18 (11–28)	0.165
Total score	138.65 ± 19.80	137.51 ± 18.63	138.06 ± 19.18 (88–191)	0.609

**Table 3:** Distribution of physical activity in men and women within age groups of 40–49 and 50–60 years.

IPAQ parameters	Mean ± SD of IPAQ parameters in the two age groups (MET min/week)					
	40–49 years			50–60 years		
	Female (*N*=79)	Male (*N*=66)	*p* value	Female (*N*=75)	Male (*N*=81)	*p* value
Walking	755.9 ± 974.5	4033.3 ± 40.39.2	<0.001	924.9 ± 834.4	2901.4 ± 3145.8	<0.001
Moderate activity	1889.1 ± 2112.9	2850.3 ± 3383.1	0.039	1782.4 ± 2602.0	4395.3 ± 5650.7	<0.001
Vigorous activity	209.1 ± 1008.2	992.1 ± 2397.2	0.009	336.5 ± 2026.0	1514.6 ± 3470.2	<0.001
Total physical activity	2854.1 ± 3143.0	7875.7 ± 5625.9	<0.001	3043.8 ± 3503.0	8811.3 ± 7550.6	<0.001

**Table 4:** Anthropometric variables, blood pressure, fasting blood sugar, and serum lipids profile of the participants.

Variables	Age groups		Total mean ± SD (min–max)	*p* value
	40–49 years (*N*=145)	50–60 years (*N*=156)		
BMI (kg/m^2^)	28.2 ± 4.6	28.3 ± 4.2	28.3 ± 4.4 (19.2–46.5)	0.773
Body fat percentage	32.3 ± 9.3	30.9 ± 11.2	31.6 ± 10.3 (5.1–52.2)	0.240
Waist circumference (cm)	93.4 ± 10.4	95.7 ± 8.7	94.6 ± 9.6 (70.0–123.0)	0.032
Buttock circumference (cm)	108.0 ± 8.8	107.1 ± 8.7	107.6 ± 8.7 (88.5–138.0)	0.372
WHR	0.86 ± 0.07	0.89 ± 0.06	0.88 ± 0.07 (0.71–1.09)	<0.001
WHtR	0.56 ± 0.06	0.58 ± 0.06	0.57 ± 0.60 (0.42–0.76)	0.001
Systolic blood pressure (mmHg)	121.2 ± 15.4	128.4 ± 16.9	124.9 ± 16.6 (88–186)	<0.001
Diastolic blood pressure (mmHg)	80.0 ± 9.9	81.1 ± 10.5	80.6 ± 10.3 (47–122)	0.347
FBS (md/dL)	95.5 ± 25.9	100.8 ± 34.5	98.3 ± 30.8 (60–330)	0.139
Total cholesterol (mg/dL)	212.0 ± 48.0	219.8 ± 44.9	216.1 ± 46.5 (115–457)	0.149
TG (mg/dL)	186.3 ± 138.9	162.7 ± 92.3	173.9 ± 117.0 (37–1108)	0.084
HDL (mg/dL)	55.9 ± 15.7	58.4 ± 14.1	57.2 ± 14.9 (21–117)	0.149
LDL (mg/dL)	113.2 ± 30.0	119.1 ± 26.9	116.3 ± 28.6 (47–233)	0.075
